# New insights into the ambivalent role of YAP/TAZ in human cancers

**DOI:** 10.1186/s13046-023-02704-2

**Published:** 2023-05-22

**Authors:** Juan Luo, Liang Deng, Hailin Zou, Yibo Guo, Tongyu Tong, Mingli Huang, Gengqiang Ling, Peng Li

**Affiliations:** 1 Scientific Research Center, The Seventh Affiliated Hospital of Sun Yat-sen University, No. 628 Zhenyuan Road, Shenzhen, 518107 Guangdong People’s Republic of China; 2 Department of General Surgery, The Seventh Affiliated Hospital of Sun Yat-sen University, No. 628 Zhenyuan Road, Shenzhen, 518107 Guangdong People’s Republic of China; 3 Department of Urology, Pelvic Floor Disorders Center, The Seventh Affiliated Hospital of Sun Yat-sen University, No. 628 Zhenyuan Road, Shenzhen, 518107 Guangdong People’s Republic of China; 4 Department of Neurosurgery, The Seventh Affiliated Hospital of Sun Yat-sen University, No. 628 Zhenyuan Road, Shenzhen, 518107 Guangdong People’s Republic of China; 5 Guangdong Provincial Key Laboratory of Digestive Cancer Research, The Seventh Affiliated Hospital of Sun Yat-sen University, No. 628 Zhenyuan Road, Shenzhen, 518107 Guangdong People’s Republic of China

**Keywords:** Hippo pathway, YAP/TAZ, Oncogene, Tumor suppressor, Targeted therapy

## Abstract

Hippo signaling was first identified in Drosophila as a key controller of organ size by regulating cell proliferation and anti-apoptosis. Subsequent studies have shown that this pathway is highly conserved in mammals, and its dysregulation is implicated in multiple events of cancer development and progression. Yes-associated protein (YAP) and transcriptional coactivator with PDZ-binding motif (TAZ) (hereafter YAP/TAZ) are the downstream effectors of the Hippo pathway. YAP/TAZ overexpression or activation is sufficient to induce tumor initiation and progression, as well as recurrence and therapeutic resistance. However, there is growing evidence that YAP/TAZ also exert a tumor-suppressive function in a context-dependent manner. Therefore, caution should be taken when targeting Hippo signaling in clinical trials in the future. In this review article, we will first give an overview of YAP/TAZ and their oncogenic roles in various cancers and then systematically summarize the tumor-suppressive functions of YAP/TAZ in different contexts. Based on these findings, we will further discuss the clinical implications of YAP/TAZ-based tumor targeted therapy and potential future directions.

**Graphical Abstract:**

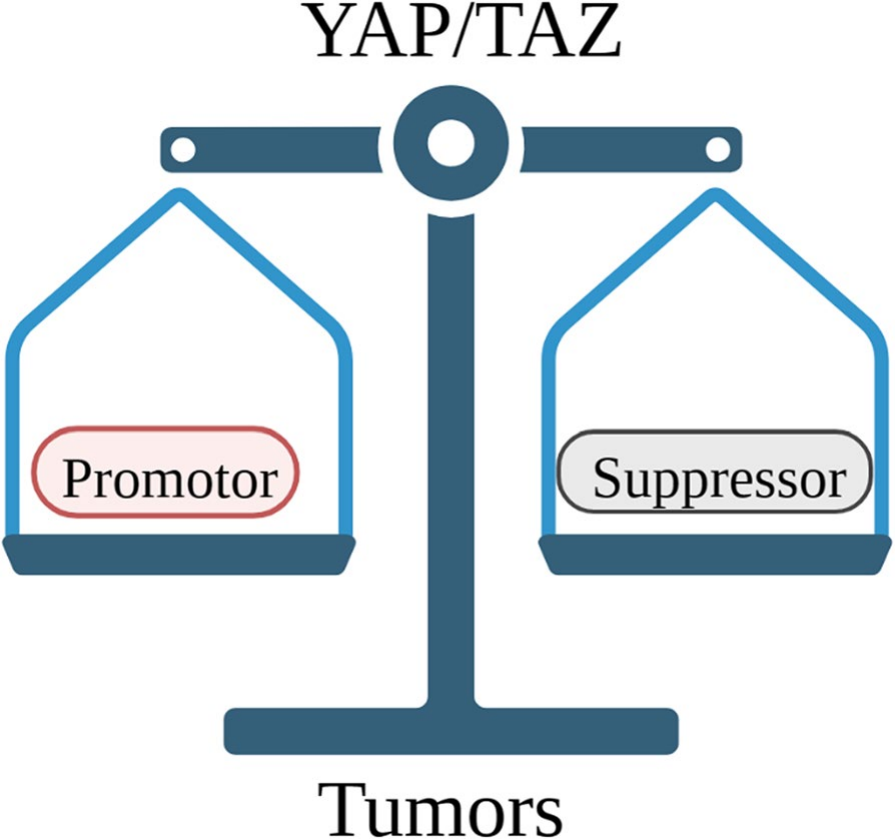

## FACTS

1. Dysregulation of Hippo signaling is implicated in multiple events of cancer development and progression.
2. YAP/TAZ overexpression or activation is sufficient to induce tumor initiation, growth and progression, as well as drug resistance.
3. YAP/TAZ exert a tumor-suppressive function in a context-dependent manner.
4. Caution should be taken when targeting Hippo signaling in clinical trials in the future.

## Open questions

1. YAP/TAZ are double-edged swords with therapeutic potential for cancers. When and where is YAP/TAZ activation more beneficial than their inhibition? Additionally, in which stage of tumor progression and for what kind of cancer types?
2. YAP/TAZ activity is essential for normal embryonic development, tissue homeostasis and regeneration. How can toxicity and negative effects on normal tissues be avoided by targeting Hippo-YAP/TAZ signaling? Targeting YAP/TAZ upstream regulators, downstream effectors, or themselves?

## Background

**Fig. 1:**
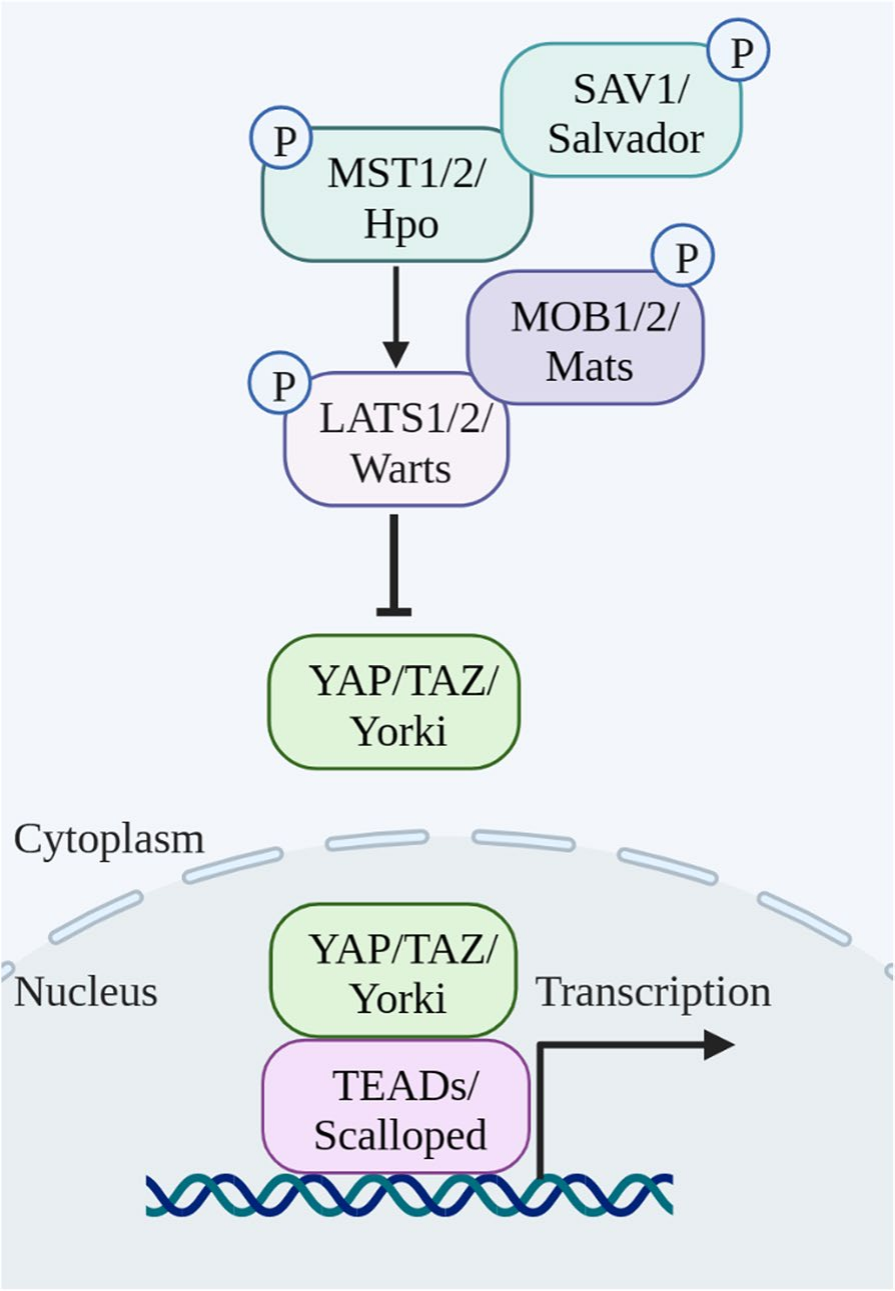
Schematic overview of the Hippo-YAP/TAZ signaling in Drosophila and Mammals. The core components of this signaling include the core kinase cascade (MST1/2/Hpo and LATS1/2/Warts), adaptors (SAV/Salvador and MOB1/2/Mats), and the downstream effectors YAP/TAZ/Yorki. The Hippo kinase cascades (MST-LATS)-mediated phosphorylation is essential for YAP/TAZ activity in the nucleus, where they interact with TEADs (Scalloped) to induce the transcription of downstream target genes

Hpo is a Ser/Thr kinase that can inhibit cell proliferation and organ growth by activating Wts kinase [1]. The Hippo pathway was initially named based on the overgrowth phenotype caused by *Hpo* mutation in Drosophila [2, 3]. Subsequently, Yorkie (Yki) was identified to function as the downstream effector of the Hpo-Wts kinase cascade, as its overexpression can phenocopy the overgrowth of *Hpo* or *Wts* loss-of-function (LOF) mutant flies [1]. Owing to these findings, the Hippo pathway is defined as a key controller of organ size in Drosophila by regulating cell proliferation and anti-apoptosis. In mammals, this pathway is highly conserved. Specifically, mammalian STE-like (MST) protein kinases (Hpo orthologs) can phosphorylate and activate large tumor suppressor (LATS) kinases (Wts orthologs), which in turn phosphorylate YAP/TAZ (Yki orthologs), leading to their cytoplasmic retention and degradation (Fig. 1) [4, 5]. Accordingly, decreased YAP/TAZ activity has been shown to inhibit cell hyperproliferation and organ overgrowth [6].

**Fig. 2:**
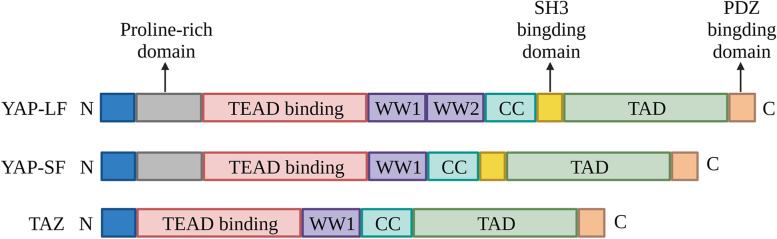
Schematic representation of YAP/TAZ proteins. There are two major isoforms of YAP protein: the short isoform (SF) contains one WW-domain (WW1), whereas the long isoform (LF) contains two WW-domains (WW1 and WW2). TAZ is represented by a unique isoform with a single WW domain. In addition, compared with YAP protein, the SH3-binding domain and the proline-rich region are absent in TAZ protein. CC: coiled coil, TAD: transcriptional activation domain

YAP/TAZ, as the final effectors of the Hippo pathway, are two highly similar proteins that show approximately 40% amino acid conservation and share several structural features [7]. For example, both contain an N-terminal domain for interaction with TEA domain (TEAD) family transcription factors, WW domains for mediating protein‒protein interactions, a C-terminal transcriptional activation domain (TAD) with a leucine zipper motif, and a PDZ-binding motif [8]. However, YAP, but not TAZ, also contains an SH3-binding motif and an N-terminal proline-rich region, which are required for the interaction with proteins containing an Src homology 3 (SH3) domain and heterogeneous nuclear ribonuclear proteins, respectively (Fig. 2) [7, 8]. Due to the lack of a DNA-binding domain, YAP/TAZ usually act as transcription coregulators (coactivator or corepressor) that need to cooperate with other DNA-binding factors to exert their functionally relevant transcription. Typically, TEAD family transcription factors (scalloped orthologs) have been proven to be the prime mediators of YAP/TAZ-associated functions, particularly in tumorigenesis [9–11]. In addition, many more YAP/TAZ-interacting partners have also been identified to modulate YAP/TAZ-associated transcriptional programs in a context-dependent manner. For additional reference, the context-dependent transcriptional regulation of YAP/TAZ in cancer and stem cells has been recently reviewed elsewhere [12, 13].

In the last two decades, accumulating evidence has demonstrated that either Hippo kinase inactivation or YAP/TAZ activation is implicated in tumor development and progression, including tumor initiation, recurrence, metastasis and therapy resistance [14]. In the meantime, many efforts are being made to develop feasible strategies for tumor treatment by targeting this pathway. However, emerging evidence reveals that the roles of YAP/TAZ in cancers are context dependent. Typically, in different contexts and tumor types, YAP/TAZ have both tumor-promoting and tumor-suppressive functions. Therefore, clarifying their functions under different conditions is of great significance for precision cancer therapy. In our current study, we will first present an overview of the dysregulation of YAP/TAZ in human cancers. Then, we systematically summarize the oncogenic roles and tumor-suppressive roles of YAP/TAZ in different contexts, as well as their underlying molecular mechanisms. Based on these ambivalent roles of YAP/TAZ in cancers, we finally discuss the clinical implications of YAP/TAZ-based tumor targeted therapy and potential future directions.

## Dysregulation of YAP/TAZ in human cancers

**Fig. 3:**
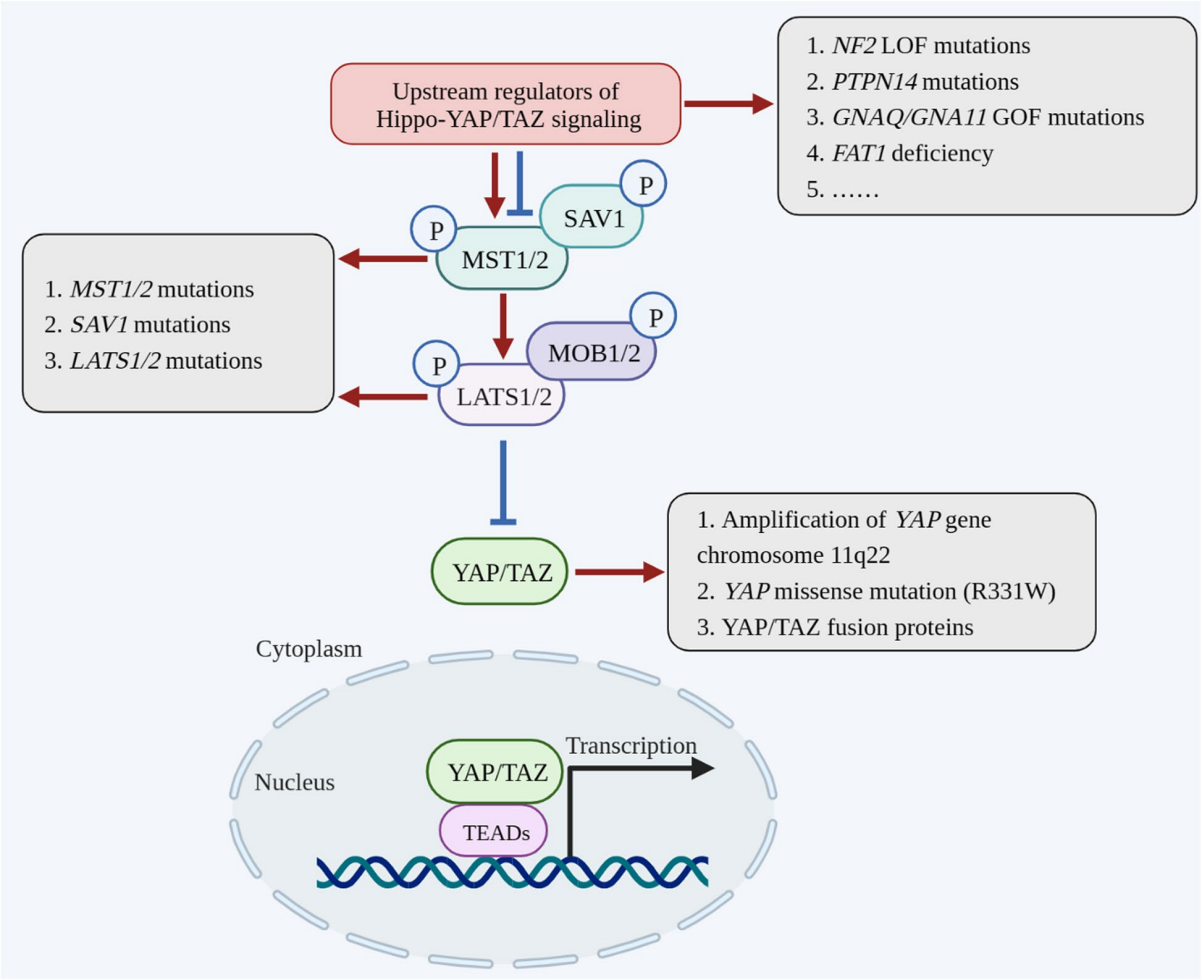
Overview of the gene mutations of Hippo core components identified in human cancers. The mutations of Hippo pathway components identified in human cancers have been highlighted in blue dialogue balloons

Given the evidence that *Yki* overexpression in flies causes cell hyperproliferation, defective apoptosis, and tissue overgrowth [1], it was thus considered to be an oncogenic protein. Indeed, extensive studies have subsequently revealed that YAP/TAZ are frequently amplified or activated in human cancers (Fig. 3). For instance, the amplification of chromosome 11q22 (where the *YAP* gene resides) is reported in multiple human cancers, including liver and breast cancers [15, 16]. Subsequent studies validated that YAP was required for sustaining the rapid growth of amplicon-containing liver cancer [15], while its overexpression in human mammary epithelial cells could induce malignant transformation [16]. In addition, *YAP* gene amplification has also been found in a subset of human hedgehog-associated medulloblastomas and esophageal squamous cell carcinomas [17, 18]. Apart from whole gene amplification, a familial inheritance point mutation (R331 W) of *YAP* was identified as an allele predisposed for lung adenocarcinoma. YAP protein carrying this mutation was shown to increase the colony-formation ability and invasion potential of lung cancer cells [19]. Moreover, *YAP/TAZ* gene fusions have recently been reported in a series of human cancers, such as *YAP-TFE3 *[20] and *TAZ-CAMTA1* gene fusions in epithelioid hemangioendothelioma [21, 22]. Many of these *YAP/TAZ* fusion transcripts are sufficient to induce tumor formation in mouse models.

With the validation of the oncogenic roles of YAP/TAZ, researchers are trying to identify the upstream regulators that are responsible for initiating the Hippo pathway and aberrant YAP/TAZ activity in various cancers. To date, multiple upstream regulators, including mechanical cues, cell polarity and cell‒cell adhesion, as well as growth factors and stress signals, have been identified to transmit various extracellular and intracellular signals to YAP/TAZ, thereby mediating their functional outputs [13]. Therefore, dysregulation of these regulators could also lead to YAP/TAZ activation for tumorigenesis (Fig. 3). For example, G protein-coupled receptors (GPCRs) represent a large family of cell surface receptors that can transmit diverse extracellular signals to the Hippo-YAP/TAZ pathway [23]. Somatic mutations of *GNAQ* or *GNA11* have been identified in approximately 83% of uveal melanoma (UM), the most common primary malignancy arising within the adult eye [24]. Multiple studies have revealed that hyperactivated YAP activity is responsible for the cell proliferation and tumor growth of *GNAQ/GNA11*-associated UM, and inhibition of YAP activity with verteporfin can reduce UM growth in a mouse model [25, 26]. In addition, *FAT1* is one of the most frequently mutated genes in human cancers [27, 28]. Pastushenko et al. found that *FAT1* deficiency can accelerate tumor initiation and malignant progression in skin squamous cell carcinoma and lung tumors by promoting a hybrid epithelial-to-mesenchymal transition (EMT) phenotype [29]. Further study revealed that YAP nuclear translocation was responsible for the phenotype induced by the loss of FAT1 [29]. Moreover, recent pancancer studies by The Cancer Genome Atlas Research in 9125 tumor samples have revealed that Hippo pathway components are widely altered in human cancers, such as downregulation of *NF2*, *FAT1*, *TAOK1-3*, *WW45*, and *LATS1/2 *[30, 31]. Taken together, these studies highlight that targeting YAP/TAZ represents an attractive therapeutic option for tumors with a dysregulated Hippo pathway.

## The tumor-promoting roles of YAP/TAZ in human cancers

**Fig. 4:**
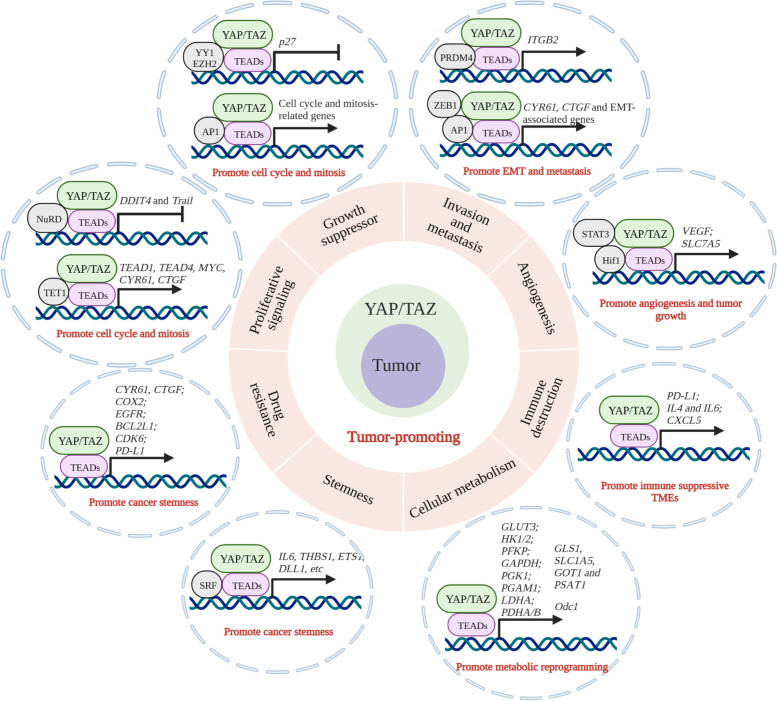
The tumor-promoting roles of YAP/TAZ and the potential mechanisms in human cancers. YAP/TAZ are implicated in the regulations of the hallmarks of cancer, including proliferation, anti-apoptosis, metastasis, drug resistance, stemness, metabolic reprogramming, angiogenesis, and microenvironment remodeling

Since the identification and characterization of the key components, as well as the signal transduction process for the Hippo pathway, researchers have been working on clarifying the detailed molecular mechanisms underlying the roles of YAP/TAZ in development and disease, especially in human cancers. To date, both in vitro and in vivo studies have demonstrated that YAP/TAZ are involved in multiple events through tumorigenesis and progression of human malignancies, including tumor growth and metastasis, drug resistance, tumor microenvironment regulation, angiogenesis, and cancer stem cell self-renewal. Meanwhile, the molecular mechanisms underlying these processes have also gradually been elucidated. In this section, we will discuss the detailed molecular mechanisms underlying the tumor-promoting functions of YAP/TAZ (Fig. 4).

### YAP/TAZ-mediated cell proliferation, anti-apoptosis, migration and invasion

Uncontrolled cell proliferation and resistance to cell death are hallmarks of cancers [32]. The initial study in Drosophila reported that *Yki* overexpression induces cell hyperproliferation and reduces cell apoptosis by controlling the cell cycle regulator *cycE* and the cell death inhibitor *diap1*, respectively [1]. Similarly, in mammalian cells, overexpression of constitutively activated YAP (S127/397A) results in increased cell proliferation and loss of cell contact-dependent inhibition [4, 33]. In vivo studies also showed that YAP activation increased liver size and caused aberrant tissue expansion in mice [34, 35]. Due to this evidence, YAP thus represents a central regulator that coordinates cell proliferation and organ growth, as well as tumorigenesis. In these processes, TEAD family transcription factors have been demonstrated to be essential for mediating YAP-associated transcriptional function [9–11]. Furthermore, *CTGF* and *BIRC5* are identified as the direct target genes of YAP-TEAD that regulate cell growth and anti-apoptosis, respectively [36]. More interestingly, Kim et al. recently showed that PRDM14-mediated transcriptional upregulation of *CALM2* and *SLC2A1* in colon cancers can rescue YAP suppression to sustain cell proliferation and survival [37], indicating the dominant roles of CALM2 and SLC2A1 in mediating YAP-associated cell proliferation and tumorigenesis.

In addition to the abovementioned mechanisms, emerging evidence has also revealed that YAP/TAZ and AP-1 family members form a complex that synergistically activates target genes directly involved in the control of S-phase entry and mitosis [38, 39]. Genome-wide association analysis showed that this complex mostly occurred at the distal enhancers that contacted target promoters through chromatin looping [40, 41]. Moreover, both overexpressed YAP/TAZ could form liquid‒liquid phase-separated bodies on these enhancers, which were required for the transcription of YAP-specific proliferation genes [42–44]. Recently, the Pan group also reported that the 5-methylcytosine dioxygenase TET1 was a direct transcriptional target of YAP in the liver, which in turn directly interacted with YAP/TEAD to cause regional DNA demethylation, histone H3K27 acetylation and chromatin opening [45]. In addition to its function as a transcription coactivator, the YAP/TAZ-TEAD complex is also able to recruit the NuRD complex to deacetylate histones and repress the expression of *DDIT4* and *Trail*, which are necessary for mTORC1 activation and cell survival, respectively [46]. In addition, YAP/TAZ can recruit EZH2 to the genome to repress the expression of the cell cycle kinase inhibitor gene *p27* or tumor suppressor gene *TGFBR2*, thereby overcoming cell‒cell contact inhibition and promoting cell hyperproliferation in human cancer cells [47, 48]. Overall, all these studies extended the previous knowledge on the transcriptional regulation of cell proliferation and tumorigenesis by YAP/TAZ, which provides a host of new therapeutic targets for tumors.

In addition to promoting cell proliferation and tumor growth, increased YAP/TAZ activity is also able to induce the EMT of normal mammary epithelial cells in vitro [4, 16, 49], as well as in vivo tumor metastasis [50, 51]. Mechanistically, the YAP-ZEB1 interaction can shift ZEB1 from a transcriptional repressor to an activator, thereby stimulating the transcription of cancer aggressiveness-associated genes [52]. Further investigations validated that ZEB1 formed a transactivation complex by cooperating with AP-1 family factors and YAP/TEAD to mark the most aggressive subtypes of breast cancer [53]. Similarly, Liu et al. also showed that YAP/TEAD-AP1 co-occupies active enhancer or promoter regions in diverse cancer cells to drive a core set of downstream target genes and coordinate cancer cell migration and invasion [54]. All these studies thus highlight the central role of the YAP/TEAD-AP1 complex in tumor cell growth and metastasis. In addition, YAP-PRDM4 interaction-mediated *ITGB2* expression was also found to be required for cell invasion in metastatic prostate cancer [55], while YAP-induced expression of *ARHGAP29* could promote tumor cell migration by suppressing the RhoA-LIMK-cofilin pathway [56]. Taken together, these studies demonstrated that YAP/TAZ provided a versatile platform on the genome to coordinate gene transcription and cell proliferation and metastasis, basically by recruiting different transcription factors or epigenetic modifiers.

### YAP/TAZ-mediated drug resistance in tumor therapy

Drug resistance in tumor cells is one of the major reasons for therapeutic failure. YAP/TAZ have been implicated in therapy resistance in various cancers. For example, TAZ-mediated expression of *CYR61* and *CTGF* has been reported to be an important modulator of the response to Taxol in breast cancer [57, 58]. Specifically, TAZ overexpression conferred resistance to Taxol, while TAZ deletion sensitized breast cancer cells to doxorubicin, suggesting that inhibition of TAZ activity could contribute to overcoming chemotherapy resistance in breast cancer [57, 58]. In addition, YAP-induced *COX-2* expression in colorectal cancer and *EGFR* expression in esophageal carcinoma were also revealed to be associated with increased Taxol resistance and resistance to 5-FU and docetaxel, respectively [59, 60]. Therefore, downregulation of COX-2 or EGFR in YAP-induced cancer cells could increase chemosensitivity [53, 54]. Targeted therapy, including using small molecules and monoclonal antibodies, has opened a new era of cancer treatment and significantly improved the prognosis of patients. However, primary or acquired resistance to these drugs is also frequently encountered. Mutations in *RAF* or *RAS* are frequently identified in human cancers, and patients with these mutations are eligible for treatment with BRAF or MEK inhibitors [61]. Both YAP overexpression and YAP-associated transcriptional signatures have been linked to poor prognosis in patients treated with BRAF inhibitors or BRAF and MEK inhibitor combinations [62, 63]. In particular, YAP-mediated *BCL2L1* expression has been demonstrated to contribute to BRAF inhibitor resistance in different *BRAF*-mutated cancer cells [62]. In addition, YAP/TAZ-regulated actin polymerization and actomyosin tension could also confer BRAF inhibitor resistance to melanoma cells [64]. Targeted inhibition of CDK4/6 has shown efficacy in the treatment of patients with estrogen receptor-positive (ER+) metastatic breast cancer [65]. Li et al. reported that *FAT1* and *RB1* LOF mutations are linked to drug resistance in breast cancer patients treated with CDK4/6 inhibitors [65]. Further study showed that YAP-induced *CDK6* upregulation was responsible for CDK4/6 inhibitor resistance, highlighting the central role and clinical value of CDK6 in breast cancer therapy [65]. Anti-PD-1/PD-L1 therapy has shown promising clinical outcomes in the treatment of many cancer types, whereas resistance is common in solid tumors. Yu et al. reported that YAP-mediated phase separation and transcription can contribute to interferon-γ-dependent immunotherapy adaptive resistance, which leads to tumor survival and immunotherapy resistance [66]. Taken together, these studies highlight that YAP/TAZ-mediated transcriptional outputs play essential roles in drug resistance in tumor therapy. Therefore, targeting YAP/TAZ or their transcriptional outputs in different cancers may serve as a rational treatment regimen to overcome drug resistance.

### YAP/TAZ-mediated regulation of tumor stemness

Cancer stem cells (CSCs) are defined as a part of the cell population, specifically endowed with self-renewal ability in vitro and tumor initiation potential in vivo. An early study in adult organs showed that YAP is highly expressed in the undifferentiated progenitor/stem cell compartment, and YAP activation expands these cell populations and leads to organ overgrowth [34]. Subsequently, YAP was also found to be elevated during induced pluripotent stem cell reprogramming, and its knockdown led to a loss of embryonic stem cell pluripotency [67]. These studies thus supported that YAP might function as a stemness regulator. Indeed, Cordenonsi et al. subsequently showed evidence that TAZ gain-of-function (GOF) endows non-CSCs with self-renewal abilities, tumorigenicity and migratory activities [68], while TAZ LOF in breast CSCs severely impairs metastatic colonization and chemoresistance [58]. In addition, Kim et al. also found that YAP activation could induce a large number of mammary stem cell signature genes, such as *IL6*, by cooperating with the transcription factor SRF [69]. Furthermore, SRF-YAP-IL6 signaling was found to be enriched in basal-like breast cancer patients and required for maintaining cancer stemness [69]. Other cancer stemness-related proteins, including OCT4, SOX2 and SOX9, were also found to be direct transcriptional targets of YAP/TAZ in multiple cancer types. Typically, YAP-driven *SOX9* expression is a critical event in the acquisition of CSC properties in esophageal and pancreatic cancer cells, suggesting that YAP inhibition may offer an effective means of therapeutically targeting the CSC population [70, 71]. More interestingly, Schaal et al. reported that nicotine could induce *SOX2* through a YAP/E2F1/OCT4 signaling axis, which accounted for the nicotine-mediated promotion of stemness in lung cancer [72]. Combined together, these findings support that targeting YAP/TAZ-dependent cancer stemness represents an attractive therapeutic strategy for cancer treatment.

### YAP/TAZ-mediated metabolic reprogramming of tumor cells

Dysregulation of metabolic pathways is one of the hallmarks of cancer. Given the dominant roles of YAP/TAZ in tumor cell survival and growth, they have indeed been revealed to influence cancer progression by regulating tumor metabolism, including glucose, fatty acid, and amino acid metabolism [73]. For instance, glucose transporter 3 (GLUT3) is frequently overexpressed in tumors; meanwhile, it has been identified to be a direct transcriptional target of YAP [74]. Moreover, a subset of glioblastomas exhibited an addiction to GLUT3, which was sensitive to agents disrupting the YAP-TEAD interaction [74]. This study thus highlighted the essential role of YAP-mediated glucose uptake in tumor cell growth. In addition, YAP/TAZ are also involved in the metabolic reprogramming of tumor cells to coordinate the environmental conditions and tumor growth. Typically, cancer cells are inclined to produce ATP through glycolysis instead of oxidative phosphorylation even under aerobic conditions, which is referred to as the Warburg effect. YAP/TAZ have been reported to directly induce the expression of several genes involved in glycolysis in different cancer types, including *HK1*, *HK2*, *PFKFB4*, *PFKP*, *PKM2*, *GAPDH*, *PGK1*, *PGAM1*, *LDHA*, *PDHA1*, and *PDHB* [74–76]. Moreover, YAP/TAZ activation can repress mitochondrial respiration, oxidative phosphorylation, and oxidative stress-induced cell death [75]. In addition to aerobic glycolysis, glutamine is of great importance for maintaining cellular hyperplasia or malignancy [77]. Multiple studies have reported that many glutamine-metabolizing enzymes are the transcriptional targets of YAP-TEAD in cancer cells, including *GLS1*, *SLC1A5*, *GOT1* and *PSAT1* [78, 79]. These studies supported that YAP/TAZ-mediated glutaminolysis represents a novel tumorigenesis mechanism and a therapeutic target. Recently, the Pan group reported that YAP/TAZ-mediated *ODC1* transcription and polyamine biosynthesis could further activate the eIF5A hypusination-LSD1 axis, which coordinated metabolic and epigenetic reprogramming and tumorigenesis [80]. Taken together, these studies highlight that targeting YAP-mediated metabolic reprogramming in cancers also represents a very attractive treatment strategy.

### YAP/TAZ-mediated tumor angiogenesis

Tumor-associated angiogenesis is critically important for continued tumor growth and metastasis. Extensive studies have confirmed that vascular endothelial growth factor (VEGF) is a major driver of blood vessel formation in both normal tissues and cancers. Emerging evidence has shown that YAP/TAZ act as central mediators of VEGF signaling to mediate angiogenesis [81–83]. Particularly, in tumor cells, Ma et al. reported that YAP/TAZ can complex with HIF-1α to promote *VEGF* expression in response to hypoxia, thereby facilitating angiogenesis and tumor growth [84, 85]. Recently, Shen et al. discovered that YAP/TAZ are nuclear localized and activated in endothelial cells (ECs) of metastatic patient colorectal cancers [86]. Further investigation showed that YAP/TAZ associated with STAT3 in tumor-associated ECs to enhance TEAD-associated transcription [87, 88]. Pharmacological inhibition of YAP/TAZ suppressed tumor angiogenesis and tumor progression in both cancer cells and mouse models. These studies suggested that YAP/TAZ activation in both cancer cells and tumor-associated ECs could contribute to tumor development by promoting tumor-associated angiogenesis. More recently, Ong et al. reported that endothelial nutrient acquisition was an essential regulator of YAP/TAZ-induced angiogenesis [89]. Specifically, YAP/TAZ-mediated *SLC7A5* transcription stimulated the import of amino acids and other essential nutrients, which in turn activated mTORC1 to promote angiogenic growth [89]. This study further highlighted the central role of YAP/TAZ in coordinating angiogenesis and tumor growth, as well as the therapeutic value of targeting YAP/TAZ-mediated angiogenesis.

### YAP/TAZ-mediated tumor microenvironment (TME) regulation

The TME is a complex ecosystem of various cellular elements, as well as acellular components, which synergistically potentiate tumor growth and progression. The acellular components are composed of the extracellular matrix (ECM), exosomes, and cytokines, while cellular components include fibroblasts, ECs, adipocytes, and immune cells [90]. In addition, the TME is usually characterized by acidic pH, hypoxia, increased interstitial pressure, inflammation and immunosuppression [90]. To date, accumulating studies have shown that YAP/TAZ-mediated TME remodeling plays an essential role in tumor development.

The increased rigidity of the ECM surrounding the cells has been proven to be related to abnormal cell behaviors, including cell hyperproliferation, migration and metastasis [91]. YAP/TAZ have been identified as both sensors and mediators of mechanical signals from the microenvironment, including ECM rigidity [91]. Typically, Chang et al. found that TAZ regulates the formation of an LM511 matrix by transcriptionally regulating *LMa5* expression in breast cancer [92]. The activation of LM511-integrin α6β1 signaling can further contribute to CSC properties by activating TAZ [92]. Likewise, YAP activation in cancer-associated fibroblasts can also contribute to the matrix stiffening of breast cancer, thereby promoting cancer cell growth and invasion [93]. Targeting the immune microenvironment is a hotspot in tumor immunotherapy. Typically, PD-L1 is an immune checkpoint molecule that is responsible for the interaction between tumor-infiltrating lymphocytes and cancer cells. PD-L1 in cancer cells can bind to its receptor PD-1 on T cells to suppress its antitumor activity [94]. *PD-L1* has been identified to be a direct transcriptional target of YAP/TAZ-TEAD in tumor cells [95], which thus establishes an immunosuppressive TME for YAP/TAZ-induced cancers. Tumor-associated macrophages (TAMs) are divided into M1 and M2 macrophages. YAP activation has been demonstrated to be associated with the polarization of TAMs to the M2 phenotype, thereby reducing the capacity of antigenic presentation of TAMs [96, 97]. Further investigation revealed that YAP could promote tumorigenesis of colon cancer by increasing the expression of M2-promoting IL-4 and tumor-promoting IL-6 cytokines [98]. Similarly, YAP-induced *CXCL5* upregulation in prostate cancer could attract CXCR2-expressing myeloid-derived suppressor cells, thereby blocking the immune cell response and promoting tumor progression [99]. In addition, YAP-mediated inhibition of CD4/CD8-positive cell differentiation and the activation of regulatory T cells also potentiate the immunosuppressed microenvironment to ensure the survival of tumors [100, 101]. Taken together, these discoveries support that YAP/TAZ are multifunctional regulators of tumor development by coordinating both tumor cell behaviors and TME remodeling Table 1.

**Table 1:** Summary of the oncogenic roles of YAP/TAZ in different tumor types and cellular contexts

Functions	Cancer types	Partners	Transcriptional outputs	Mechanisms	Main reference
**Cell proliferation and anti-apoptosis**	Multiple cancer cells	TEADs	*CTGF* and *BIRC5*	YAP/TAZ-mediated transcription regulates cell growth and anti-apoptosis	[9–11, 34–36]
	Multiple cancer cells	TEAD and AP-1 family members	S-phase entry and mitosis-related genes	YAP/TAZ cooperates with TEAD and AP-1 family transcription partners on enhancers to induce the transcription of YAP-specific proliferation genes	[38–41]
	Liver cancer	TEADs and TET1	*TET1*	YAP-induced the expression of *TET1* can cooperate with TEADs to facilitate the transcription of YAP/TAZ target genes	[45]
	Multiple cancer cells	TEADs and NuRD	*DDIT4* and *Trail*	YAP/TAZ-TEAD complex recruits the NuRD complex to repress the expression of *DDIT4* and *Trail*, thereby promoting mTORC1 activation and cell survival	[46]
	Multiple cancer cells	YY1 and EZH2	*p27* or *TGFBR2*	YAP/TAZ recruit EZH2 on the genome to repress the expression of *p27* or *TGFBR2*, thereby overcoming cell-cell contact inhibition and promoting cell hyperproliferation in human cancer cells	[47, 48]
**Migration and invasion**	Breast cancer	TEADs, ZEB1 and AP-1 family members	Cancer aggressiveness-associated genes	YAP/TAZ-TEAD complex cooperates with ZEB1 and AP-1 family members to directly activate the transcriptions of cancer aggressiveness-associated genes	[52–54]
	Prostate cancer	PRDM4	*ITGB2*	YAP/PRDM4-mediated *ITGB2* expression can promote cell migration	[55]
	Gastric cancer	TEADs and FOS	*ARHGAP29*	YAP promotes the expression of *ARHGAP29* to suppress the RhoA-LIMK-cofilin pathway, thereby promoting cell migration	[56]
**Drug resistance**	Breast cancer	TEADs	*CTGF* and *CYR61*	TAZ-mediated expression of *CYR61* and *CTGF* promotes the resistance to taxol and doxorubicin in breast cancer	[57, 58]
	Colorectal cancer	TEADs	*COX-2*	YAP-induced *COX-2* expression is associated with the increased taxol resistance in colorectal cancer	[59]
	Esophageal carcinoma	TEADs	*EGFR*	YAP-induced *EGFR* expression is associated with the increased resistance to 5-FU and docetaxel in esophageal carcinoma	[60]
	*BRAF-*mutated cancer cells	TEADs	*BCL2L1*	YAP-mediated *BCL2L1* expression contributes to the BRAF inhibitor resistance in different *BRAF*-mutated cancer cells	[64]
	ER + metastatic breast cancer	TEADs	*CDK6*	YAP-induced *CDK6* expression is responsible for the CDK4/6 inhibitor resistance in metastatic breast cancer	[65]
	Lung adenocarcinoma	TEADs and EP300	*MYC* and *CD155*	YAP mediated-phase separation and transcription contribute to the interferon-γ-dependent immunotherapy adaptive resistance	[66]
**Stemness regulation**	Breast cancer	TEADs and SRF	*IL6*	SRF-IL6 axis is the critical mediator of YAP-induced stemness in mammary epithelial cells and breast cancer	[69]
	Esophageal cancer	TEADs	*SOX9*	YAP regulates the transcription of *SOX9* and endows esophageal cancer cells with stem-like properties	[70]
	Pancreatic ductal adenocarcinoma	PAF1 and TEADs	*SOX9*	PAF1 cooperates with YAP/TEAD to induce the transcription of *SOX9*, and which endows pancreatic cancer cells with stem-like properties	[71]
	Lung cancer	E2F1 and OCT4	*SOX2*	YAP binding to E2F1 and/or OCT4 upregulates *SOX2* expression, thereby enhancing self-renewal of CSCs	[72]
**Metabolic reprogramming**	Glioblastoma	TEADs	*GLUT3*	YAP-mediated glucose uptake through upregulating *GLUT3* promotes tumor cell growth	[74]
	Multiple cancer cells	TEADs	Glycolysis-associated genes	YAP/TAZ-mediated transcriptions promote glycolysis and repress mitochondrial respiration, oxidative phosphorylation, as well as oxidative stress-induced cell death	[75]
	Hepatocellular carcinoma	HIF-1α	*PKM2*	Hypoxia-induced YAP/HIF-1α interaction promotes *PKM2* gene expression and accelerates glycolysis	[76]
	Multiple cancer cells	TEADs	*GLS1, SLC1A5, GOT1* and *PSAT1*	YAP/TAZ-mediated glutaminolysis promotes tumorigenesis in multiple cancer cells	[78, 79]
	Multiple cancer cells	TEADs	*ODC1*	YAP/TAZ-mediated *ODC1* transcription promotes polyamine biosynthesis and the polyamine-eIF5A hypusination-LSD1 axis to drive tumorigenesis	[80]
**Tumor angiogenesis**	Multiple cancer cells	HIF1α	*VEGF*	YAP/TAZ can complex with HIF1α to promote *VEGF* expression and tumor angiogenesis and growth	[84, 85]
	Tumor-associated endothelial cells	STAT3	*VEGF and TNFα*	YAP/TAZ associate with STAT3 in tumor-associated endothelia cells to enhance TEAD-associated transcription and angiogenesis	[86–88]
	Tumor-associated endothelial cells	TEADs	*SLC7A5*	YAP/TAZ-mediated *SLC7A5* transcription stimulates the import of amino acids and other essential nutrients to promote angiogenic growth	[89]
**Tumor microenvironment regulation**	Breast cancer	TEADs	*LM511*	TAZ regulates the matrix formation through transcriptionally regulating *LMa5* expression, and which in turn contributes to the CSC-properties by activating TAZ	[92]
	Breast cancer	TEADs	*ANLN* and *DIAPH3, MYL9*	YAP regulates the expression of several cytoskeletal regulators and contributes to the matrix stiffening of breast cancer	[93]
	Multiple cancer cells	TEADs	*PD-L1*	YAP/TAZ-induced *PD-L1* expression facilitates the establishment of an immunosuppressive TME in human cancers	[95]
	Colon cancer	/	*IL4/6*	YAP promotes tumorigenesis of colon cancer through increasing the expression of M2-promoting IL-4 and tumor-promoting IL-6 cytokines	[98]
	Prostate cancer	TEADs	*CXCL5*	YAP-induced *CXCL5* upregulation in prostate cancer can attract CXCR2-expressing myeloid-derived suppressor cells, thereby blocking immune cell response and promoting tumor progression	[99]

## The tumor-suppressive roles of YAP/TAZ in human cancers

**Fig. 5:**
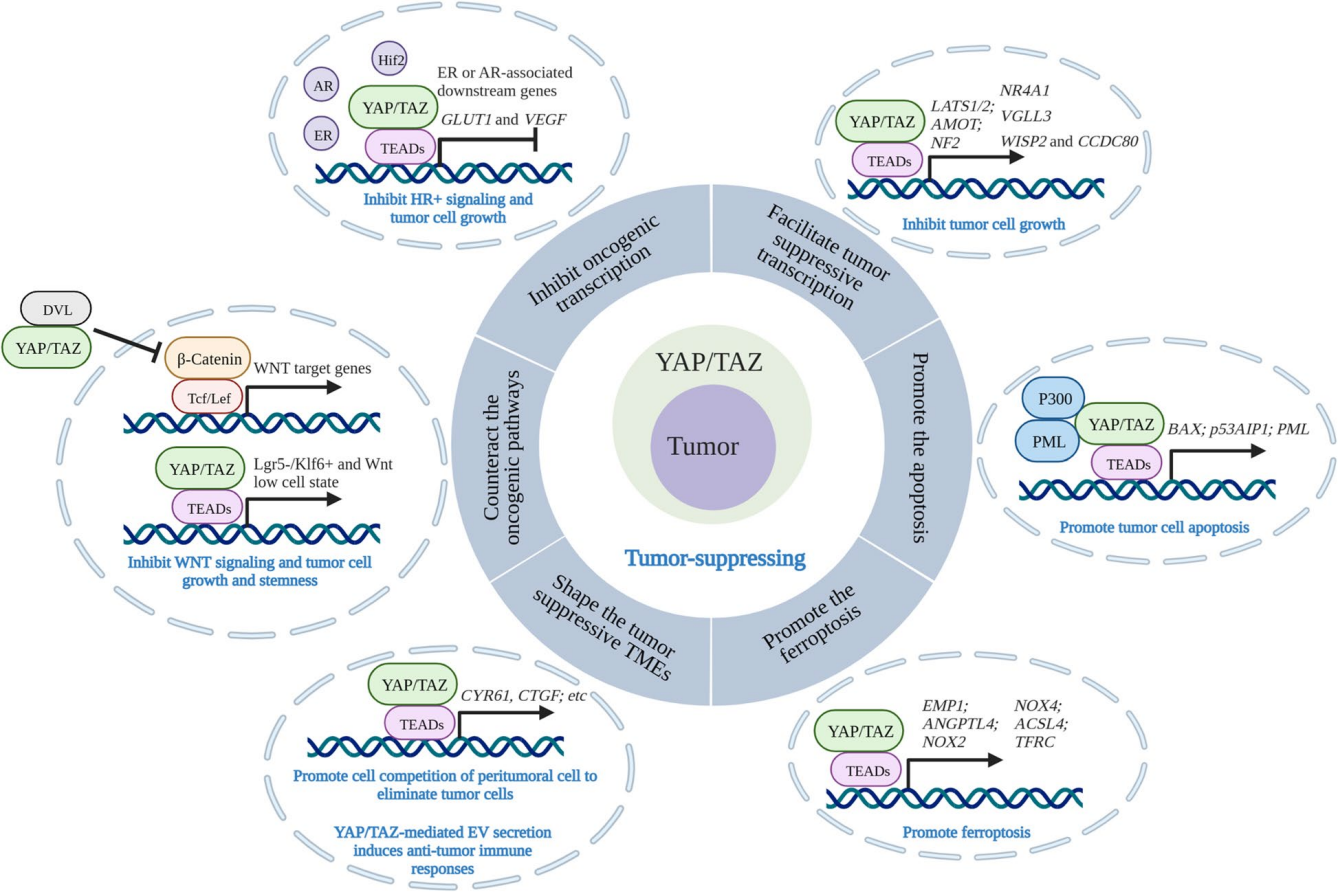
The tumor-suppressive roles of YAP/TAZ and the potential mechanisms in human cancers. YAP/TAZ exert a tumor-suppressive function in a context-dependent manner through different molecular mechanisms

Although YAP/TAZ overexpression or activation has been proven to promote tumor progression via multiple mechanisms, accumulating evidence shows that YAP/TAZ also exert tumor-suppressive functions in a context-dependent manner. Typically, 11q22, the *YAP* gene-residing region, is frequently lost of heterozygosity (LOH) in some breast cancers [102, 103]. Further investigations revealed that LOH of the 11q22 amplicon was associated with the invasive subtype and poor survival in breast tumors [104]. Consistently, YAP knockdown in breast cancer cells increased migration and invasion abilities, inhibited the response to Taxol and enhanced tumor growth in nude mice [105]. Moreover, recent in vivo studies also revealed that depletion of YAP in breast cancer cells led to significantly more lung metastasis [106]. All these studies thus indicated a potent tumor-suppressive role of YAP/TAZ. More interestingly, Pearson et al. recently reported that solid tumors can be classified into two categories, named “YAP on”, in which YAP is highly expressed and behaves as an oncogene, and “YAP off”, in which its expression is silenced and it behaves as a tumor suppressor [107]. “YAP off” solid cancers are usually RB1 deficient, including retinoblastoma, small cell lung cancer, and neuroendocrine prostate cancer [107]. In “YAP off” solid cancers, re-expression of YAP to activate genes belonging to the integrin/ECM/adhesion pathway can induce cytostasis [107]. In this section, we mainly aim to systematically summarize the emerging tumor-suppressive roles of YAP/TAZ and their underlying molecular mechanisms in different contexts (Fig. 5).

### YAP/TAZ interfere with the transcriptional program of key oncogenic factors

Hormone-associated tumors, such as ER + breast cancer and androgen receptor-positive (AR+) prostate cancer, are mainly dependent on hormone receptor (HR) signaling to sustain tumor cell growth and survival. Therefore, interfering with HR-related functions and signal transduction processes is the mainstay treatment for these cancers. An early study reported that the YAP-TEAD complex and ERα could co-occupy the superenhancer regions of ERα-associated target genes to mediate estrogen-associated transcription and breast cancer growth [108]. However, a recent study revealed that YAP was more likely to play a tumor-suppressive role in ER + BC. Specifically, the YAP-ER association could compete with ERα for binding to TEAD, which led to the dissociation of ERα from its target sites and subsequent degradation [109]. In the same group, they also identified that YAP acted as a context-dependent tumor suppressor in AR + prostate cancer by antagonizing TEAD-mediated AR signaling [110]. Similarly, the YAP-TEAD interaction was also found to be a competitor for the TEAD-Hif-2α complex in clear cell renal cell carcinoma (ccRCC) [111]. Increased nuclear YAP reduced ccRCC tumor growth by decreasing HIF-2α target gene expression, including *GLUT1* and *VEGF* [111]. All these studies indicated a general tumor-suppressive mechanism of YAP/TAZ through interference with the transcriptional program of key oncogenic factors, especially for HR + cancers. In addition, TAZ expression was lower in hematological malignancies, while its high expression was correlated with better patient outcomes [112]. Further investigations showed that TAZ elicited an antitumorigenic function by repressing *MYC* expression and its transcriptional program [112]. Therefore, inhibition of Hippo signaling in these contexts may be a more rational strategy for cancer therapy.

### YAP/TAZ facilitate the transcriptional program of tumor suppressors

Although YAP/TAZ usually promote the expression of genes related to tumor promotion, early studies also showed that YAP/TAZ activation induced the transcription of their negative regulators, including LATS1/2, AMOT, and NF2, to establish a negative feedback loop and prevent tumorigenesis [113, 114]. Recently, He et al. identified *NR4A1* as a novel target of YAP that mediates the proapoptotic and antitumor effects of the Hippo pathway [115]. Specifically, YAP-mediated *NR4A1* transcription could promote YAP degradation and inhibit YAP-induced liver regeneration and tumorigenesis [115]. Overall, these studies highlighted a negative feedback regulatory mechanism for YAP/TAZ in organ growth.

Vestigial-like protein 4 (VGLL4) has been reported to inhibit YAP-TEAD transcriptional activity by displacing YAP from TEAD [116–118]. Ma et al. recently reported that *VGLL3* was a direct transcription target of YAP-TEAD in ER + BC [119]. YAP-induced *VGLL3* could further compete with YAP/TAZ for binding to the TEAD transcription factor and then recruit the NCOR2/SMRT repressor to the superenhancer of the *ESR1* gene, leading to epigenetic alteration and transcriptional silencing [119]. This study thus revealed another mechanism for YAP-associated tumor suppressor function in ER + breast cancer. Trichorhinophalangeal syndrome 1 (TRPS1) is commonly overexpressed in breast cancer. Elster et al. reported that TRPS1 is a potent repressor of YAP-dependent transactivation [120], while YAP is found to induce the expression of genes related to immunosurveillance in this context [120]. In addition, Huang et al. reported that digitoxin can suppress human lung squamous cell carcinoma growth both in vitro and in vivo by attenuating YAP phosphorylation and promoting YAP nuclear sequestration [121]. Further study showed that YAP activation led to excessive accumulation of reactive oxygen species by downregulating the antioxidant enzyme GPX2 [121]. This study thus highlighted a novel tumor-suppressor function of YAP via downregulation of *GPX2*, with potential implications for improving precision medicine for human lung squamous cell carcinoma. In addition, YAP activation also caused a growth inhibitory effect in mouse MC38 colon cancer cells by inducing the expression of *Wisp2* and *Ccdc80* [122]. Deletion of these two genes prevented the growth inhibitory effect of YAP activation in these cells [122]. Recently, Frost et al. showed that YAP/TAZ suppressed the growth of MCPyV-positive Merker cell carcinoma cells through TEAD-dependent transcriptional repression of *MCPyV LT* [123], further highlighting that the function of YAP/TAZ was highly dependent on their transcriptional outputs in different cancers.

### YAP/TAZ enhance susceptibility to apoptosis-inducing agents

Apoptosis is an important mechanism to eliminate oncogenesis. The p53 family proteins play an essential role in inducing cell cycle arrest or apoptosis. YAP/TAZ have been found to play a proapoptotic role by interacting with p73 (a homolog of p53), which can further induce p73-associated target genes in response to DNA damage [124, 125]. Furthermore, the YAP-p73 association is positively regulated by ABL-mediated YAP tyrosine phosphorylation at Y357 but repressed by AKT and LATS-mediated serine phosphorylation at S127 [126, 127]. Subsequently, Lapi et al. identified promyelocytic leukemia (*PML*), a tumor suppressor gene, as a direct transcriptional target of the YAP-p73 complex [128]. PML could further interact with YAP and cause PML-mediated sumoylation and stabilization of YAP, which eventually accelerated DNA damage-induced apoptosis [128]. Consistently, EGR-1 was identified to be upregulated in prostate carcinoma cells by ionizing irradiation, and it could complex with YAP to upregulate *Bax* expression, thereby enhancing the susceptibility to radiation-induced apoptosis [129]. In hematologic malignancies, including leukemias, lymphomas, and multiple myeloma, *YAP* was found to be deleted or consistently downregulated [130]. Further investigation showed that YAP activation could also trigger DNA damage-induced apoptosis in these cancers, further supporting a tumor suppressor function of YAP in hematological malignancy [130]. More interestingly, Gujral et al. recently showed that nuclear YAP enhanced gemcitabine intracellular availability in multiple human pancreatic cancer cells and tumors by downregulating the expression of multidrug transporters [131], supporting that YAP activation could also contribute to overcoming drug resistance in pancreatic cancer.

### YAP/TAZ promote ferroptosis

Ferroptosis is a new type of iron-dependent regulated cell death mechanism that can contribute to antitumor function [132] and thus represents a novel method for treating cancer. Multiple studies have demonstrated that YAP/TAZ play essential roles in regulating ferroptosis [133]. For example, TAZ is activated in both RCC and ovarian cancer (OC). Yang et al. found that TAZ activation can enhance the susceptibility of RCC to ferroptosis by inducing the expression of *EMP1* and *NOX4 *[134], while in OC, the TAZ-ANGPTL4-NOX2 signaling axis mediates cell density-regulated ferroptosis [135]. These studies thus implied that TAZ status could serve as a predictor of ferroptosis sensitivity and novel therapeutic potential for both RCC and OC. In addition, inactivation of NF2 in mesothelioma cells activates YAP, which enhances cellular sensitivity to ferroptosis [136]. Further studies showed that YAP induced ferroptosis by upregulating several ferroptosis modulators, including *ACSL4* and *TFRC* [136]. Taken together, these studies suggest that activation of YAP/TAZ-mediated ferroptosis offers an attractive strategy for cancer treatment in the future.

### YAP/TAZ shape the suppressive tumor microenvironment

The TME plays an important role in tumor growth, metastasis and drug resistance. Inactivation of the Hippo pathway or YAP activation can dramatically induce liver tumorigenesis and progression [34, 35, 137]. However, Moya et al. found that YAP/TAZ were also activated in the normal hepatocytes surrounding liver tumors and that depletion of YAP/TAZ in these cells activated tumor growth [138]. Moreover, constitutively activated YAP in peritumoral hepatocytes repressed primary liver tumor growth and melanoma-derived liver metastases [138]. This study highlighted that YAP/TAZ acted through a mechanism of cell competition from TMEs to eliminate tumor cells. Recently, Nie et al. revealed that the YAP/TAZ-CD54 axis was required for CXCR2^−^CD44^−^ tumor-specific neutrophils to suppress gastric cancer, opening a new possibility to develop neutrophil-based antitumor therapeutics [139]. In addition, tumor cells can shape their microenvironment through the secretion of cytokines, chemokines, growth factors, etc. Moroishi et al. reported that either LATS1/2 deletion or YAP/TAZ hyperactivation inhibited tumor growth due to the induction of antitumor immune responses [140]. Specifically, LATS1/2-null or YAP/TAZ-activated tumor cells could secrete nucleic acid-rich extracellular vesicles that stimulated the host TLR-MYD88/TRIF-IFN pathway to induce antitumor immunity and the eventual elimination of tumor cells [140]. This study thus indicated a new paradigm for how YAP/TAZ activation in tumor cells regulates tumor immunogenicity and has implications for targeting YAP/TAZ in cancer immunotherapy.

### YAP/TAZ counteract the key oncogenic pathway

YAP/TAZ activation is widespread in many human tumors. However, inactivation of Hippo kinases or YAP/TAZ activation is insufficient to drive the initiation of most tumors [14]. Typically, the Wnt pathway is the major driving force for homeostatic self-renewal and regeneration in the mammalian intestine. Meanwhile, constitutive activation of this pathway is also the most common event for triggering colon tumor formation [141]. Barry et al. reported that cytoplasmic YAP can restrict Wnt signaling by limiting the activity of Dishevelled, thereby inhibiting the regenerative growth of intestinal epithelia [142]. Moreover, YAP is silenced in a subset of highly aggressive and undifferentiated human colorectal carcinomas, and its reactivation restricts the growth of colorectal carcinoma xenografts [142]. This study thus highlighted that YAP was a tumor suppressor in colon cancer by interfering with Wnt signaling. Recently, the same group also reported that YAP activation could maintain gut epithelial cells in a state characterized by a wound-healing signature, with increased Kruppel-like factor 6 expression and decreased Wnt signaling [143]. In contrast, deletion of YAP favored the growth of focally induced colonic tumors [143]. This study further supported that YAP acted as a tumor suppressor, and activating the Hippo kinases represented a novel therapeutic approach for combating colorectal cancers Table 2.

**Table 2:** Summary of the tumor-suppressive roles of YAP/TAZ in different tumor types and cellular contexts

Mechanisms	Cancer types	Partners	Transcriptional outputs	Functions	Main reference
**Interfere with the transcription of key oncogenes**	ER + breast cancer	ERa	/	YAP-ER association competes with ERα for binding to TEAD and inhibits ERα-associated transcription and function	[109]
	AR + prostate cancer	AR	/	YAP-AR association competes with AR for binding to TEAD and inhibits AR-associated transcription and function	[110]
	ccRCC	TEAD-Hif-2α	*GLUT1* and *VEGF*	YAP-TEAD interaction competes with TEAD-Hif-2α complex to decrease HIF-2α target gene expression and reduce tumour growth	[111]
	Hematological malignancies	/	*MYC*	TAZ elicits an antitumorigenic and proapoptotic function by repressing *MYC* expression and its oncogenic function	[112]
**Facilitate the transcription of oncosuppressor genes**	Mammalian cells	/	*LATS1/2*, *AMOT* and *NF2*	YAP/TAZ activation induces the expressions of their negative regulators to establish a negative feedback loop between YAP/TAZ and their inhibitors	[113, 114]
	Live cancer	/	*NR4A1*	YAP promotes the pro-apoptotic and anti-tumour effects medicated by NR4A1, which in turn functions as a feedback inhibitor of YAP to promote its degradation	[115]
	ER + breast cancer	VGLL3-TEADs and NCOR2/SMRT	*VGLL3*	VGLL3-TEAD complex can recruit the NCOR2/SMRT repressor to the super-enhancer of *ESR1* gene to reduce its transcription	[119]
	Breast cancer	/	Immunosurveillance-related genes	YAP induces the expression of immunosurveillance-related genes	[120]
	Lung squamous cell carcinoma	/	*GPX2*	YAP activation leads to excessive accumulation of reactive oxygen species by downregulating the antioxidant enzyme *GPX2*	[121]
	Mouse MC38 colon cancer cells	/	*Wisp2* and *Ccdc80*	YAP activation causes a growth inhibitory effect on tumor cells	[122]
	MCPyV-positive Merker cell carcinoma	TEADs	*MCPyV LT*	YAP/TAZ suppress tumor growth through TEAD-dependent transcriptional repression of *MCPyV LT*	[123]
**Enhance the susceptibility to apoptosis-inducing agents**	Multiple cancer cell lines	p73 and PML	p73-associated target genes	YAP/TAZ cooperate with p73 to induce p73-associated target genes and accelerate tumor cell apoptosis	[124–128]
	Prostate carcinoma	EGR-1	*Bax*	YAP/TAZ enhance the susceptibility to radiation-induced apoptosis	[129]
	Hematologic malignancies	/	Apoptosis-related genes	YAP activation can trigger DNA damage-induced apoptosis	[130]
	Pancreatic cancer	TEADs	*CDA*, *CTGF* and *AMOTL2*	YAP enhances gemcitabine intracellular availability in tumors by down-regulating multidrug transporters	[131]
**Promote the ferroptosis**	RCC	/	*EMP1* and *NOX4*	TAZ activation can enhance the susceptibility of RCC to ferroptosis	[134]
	Ovarian cancer	*/*	*ANGPTL4* and *NOX2*	TAZ-ANGPTL4-NOX2 signaling axis mediates the cell density-regulated ferroptosis	[135]
	Mesothelioma cells	/	*ACSL4* and *TFRC*	YAP activation enhances cellular sensitivity to ferroptosis	[136]
**Shape the suppressive tumor microenvironment**	Liver cancer	/	*CYR61*, *CTGF* and *Ankrd1*	Activated YAP in the peritumoral hepatocytes represses the primary liver tumor growth and melanoma-derived liver metastases	[138]
	Gastric cancer	/	*CD54*	YAP/TAZ-CD54 signaling axis activation in CXCR2-CD44-tumor-specific neutrophils can suppress gastric cancer growth	[139]
	Multiple cancer cell lines	/	/	YAP/TAZ-activated tumor cells can secrete nucleic-acid-rich extracellular vesicles to induce anti-tumor immunity	[140]
**Counteract the key oncogenic pathway**	Colorectal cancer	/	*KLF6*	YAP activation counteracts the Wnt signaling to repress colorectal cancer growth	[141–143]

## The therapeutic implications of YAP/TAZ in human cancers

In most human cancers, YAP/TAZ overexpression or activation induces cancer cell proliferation, metastasis, CSC attributes, drug resistance, and TME remodeling. Targeting YAP/TAZ thus always represents a large therapeutic window for cancer treatment. These also represent a mainstream view in the field of targeted therapy of Hippo-YAP/TAZ signaling. To this end, numerous drugs targeting YAP/TAZ have been developed and proven to inhibit their nuclear localization or transcriptional activity, thereby exhibiting potent antitumor effects [144, 145]. Typically, verteporfin, an FDA-approved compound for treating macular degeneration, was initially identified to block the interaction between YAP and TEAD, thereby inhibiting tumor cell growth and metastasis both in vitro and in vivo [146]. As with verteporfin, many other compounds, such as CA3 and CPD3.1 [147–149], have also been shown to interfere with YAP/TAZ-TEAD-mediated activity, thereby inhibiting tumor cell growth. VGLL4, a vestigial-like protein 4, is found to be a tumor suppressor in human cancers via direct competition with YAP for binding TEADs [116, 150]. Therefore, a VGLL4-mimicking peptide called “super-TDU” has been designed and showed antitumor efficiency both in vitro and in vivo [116, 150]. However, the target specificity and selectivity of these drugs remain to be determined. To date, three inhibitors have entered clinical stage I [151]. Among them, ION537 is an antisense nucleotide inhibitor, and for others targeting the YAP-TEAD interaction, no chemical structure has been reported [151].

In addition, AP-1 family transcription factors are the most representative partners that can cooperate with YAP/TAZ to synergistically drive oncogenic growth in YAP/TAZ-associated cancers [40]. From this perspective, targeting AP1 directly or its regulation also offers the possibility of eliminating YAP/TAZ-driven cancers. As stated, Koo et al. have shown that chemical AP-1 inhibitors (SR-11,302 or T5224) can inhibit YAP/TAZ-mediated gene transcription and oncogenic cell growth in vitro, as well as YAP/TAZ-driven liver growth in vivo [152]. Similarly, YAP/TAZ-mediated recruitment of general transcriptional cofactors, including bromodomain-containing protein 4, CDK9 and RNA polymerase II, can also boost the expression of oncogenic growth-regulating genes [39, 153]. Therefore, targeting these factors also represents a potential therapeutic strategy for YAP/TAZ-driven cancers in the future.

In our current study, we also revealed a context-dependent role for YAP/TAZ in distinct tumors. Typically, in HR + cancers, including ER + breast cancer and AR + prostate cancer, YAP/TAZ seem to perform a tumor-suppressive function through different mechanisms. However, TEADs have always performed a tumor-promoting function in these processes. Therefore, directly targeting TEADs for the treatment of HR + cancers seems to be more straightforward. For example, multiple studies have revealed that there is a novel pocket in the center of the TEAD transactivation domain that is more accessible and druggable, which makes it possible to develop TEAD-selective compounds [154–157]. In addition, XMU-MP-1, an ATP-competitive inhibitor of both MST1 and MST2, has been demonstrated to improve live repair and regeneration in multiple mouse models through YAP activation [158]. Recently, Ma et al. also developed a potent LATS inhibitor, VT02956, which is able to reduce *ESR1* expression and the growth of ER + breast cancer cell lines and patient-derived tumor organoids [119]. Therefore, inhibition of Hippo kinase cascades might be an attractive strategy for HR + cancer treatment in the future.

## Conclusion

In this review, we have provided a comprehensive summary of the roles of YAP/TAZ in carcinogenesis. Unlike previous reviews, we also focus on the emerging tumor-suppressive roles of YAP/TAZ in various cancers. Our study thus indicated more complex roles for Hippo-YAP/TAZ signaling in different cancer types, as well as in different contexts. Based on current research, caution should be exercised when translating the results to the clinical setting in the future by targeting Hippo-YAP/TAZ signaling. To this end, we also advocate that more functional studies and mechanistic insights are needed to clarify the precise role of YAP/TAZ in specific cancer types and in distinct TMEs. For this purpose, multiple omics analysis along with the integration of human tumor organoid and patient-derived xenograft models may enable us to obtain a more in-depth and comprehensive understanding of the functions of YAP/TAZ in cancers, therefore aiding future cancer therapy strategies.

## Data Availability

The datasets used and analyzed in this study are available from the corresponding author on reasonable request.

## Abbreviations

AR+: androgen receptor-positive
CAFs: cancer-associated fibroblasts
ccRCC: clear cell renal cell carcinoma
CSC: cancer stem cell
EC: endothelial cell
ECM: extracellular matrix
EMT: epithelial-to-mesenchymal transition
ER+: estrogen receptor-positive
GLUT3: glucose transporter 3
GOF: gain-of-function
GPCRs: G protein-coupled receptors
HR: hormone receptor
LATS: large tumor suppressor
LOF: loss-of-function
LOH: loss of heterozygosity
MST: mammalian STE-like
OC: ovarian cancer
PML: promyelocytic leukemia
PD-1/PD-L1: programmed cell death 1/programmed cell death-ligand 1
ROS: reactive oxygen species
SH3: Src homology 3
TAD: transcriptional activation domain
TAMs: tumor-associated macrophages
TAZ: transcriptional coactivator with PDZ-binding motif
TEAD: TEA domain
TMEs: tumor microenvironments
TRPS1: trichorhinophalangeal Syndrome 1
UM: uveal melanoma
VEGF: vascular endothelial growth factor
VGLL: vestigial-like protein
YAP: Yes-associated protein
